# Extracellular Vesicles as Inflammatory Drivers in NAFLD

**DOI:** 10.3389/fimmu.2020.627424

**Published:** 2021-02-02

**Authors:** Akshatha N. Srinivas, Diwakar Suresh, Prasanna K. Santhekadur, Deepak Suvarna, Divya P. Kumar

**Affiliations:** ^1^ Department of Biochemistry, CEMR, JSS Medical College, JSS Academy of Higher Education and Research, Mysuru, India; ^2^ Department of Gastroenterology, JSS Medical College and Hospital, JSS Academy of Higher Education and Research, Mysuru, India

**Keywords:** exosomes, microvesicles, nonalcoholic fatty liver disease, inflammation, cytokines, immune system

## Abstract

Non-alcoholic fatty liver disease (NAFLD) is a highly prevalent chronic liver disease in most parts of the world affecting one-third of the western population and a growing cause for end-stage liver diseases such as hepatocellular carcinoma (HCC). Majorly driven by obesity and diabetes mellitus, NAFLD is more of a multifactorial disease affected by extra-hepatic organ crosstalk. Non-alcoholic fatty liver (NAFL) progressed to non-alcoholic steatohepatitis (NASH) predisposes multiple complications such as fibrosis, cirrhosis, and HCC. Although the complete pathogenic mechanisms of this disease are not understood, inflammation is considered as a key driver to the onset of NASH. Lipotoxicity, inflammatory cytokines, chemokines, and intestinal dysbiosis trigger both hepatic and systemic inflammatory cascades simultaneously activating immune responses. Over a few years, extracellular vesicles studied extensively concerning the pathobiology of NAFLD indicated it as a key modulator in the setting of immune-mediated inflammation. Exosomes and microvesicles, the two main types of extracellular vesicles are secreted by an array of most mammalian cells, which are involved mainly in cell-cell communication that are unique to cell type. Various bioactive cargoes containing extracellular vesicles derived from both hepatic and extrahepatic milieu showed critical implications in driving steatosis to NASH reaffirming inflammation as the primary contributor to the whole process. In this mini-review, we provide brief insights into the inflammatory mediators of NASH with special emphasis on extracellular vesicles that acts as drivers of inflammation in NAFLD.

## Introduction

Non-alcoholic fatty liver disease (NAFLD), a multisystem disease comprises a spectrum of histological conditions ranging from simple steatosis to non-alcoholic steatohepatitis (NASH), fibrosis, cirrhosis, and hepatocellular carcinoma (HCC) (1, 2). NAFLD linked with several extra-hepatic chronic complications in particular diabetes mellitus and cardiovascular diseases support the fact that it is a hepatic manifestation of metabolic syndrome (3). With the growing burden and rising prevalence over the last decade, studies estimate that over 25% of the population is affected by NAFLD globally with obesity and type 2 diabetes mellitus (T2DM) being the major etiologies (4, 5). This number is predicted to increase by 2–3 folds by 2030 mainly contributed by sedentary lifestyles and high-calorie diet intake (6, 7). Although steatosis is benign, 7%–30% of affected patients develop NASH over time, which is the major risk factor for cirrhosis and HCC. NAFLD being the common cause of the chronic liver disease is also becoming the leading cause of liver transplantation worldwide (8, 9).

The pathogenic mechanisms of development and progression of the disease have evolved from simple “two-hit” to complex “multiple-hit” hypothesis in which various factors and inter-organ crosstalk synergistically acts providing solid evidence to NAFLD as a multifactorial condition (10). The fatty liver (NAFL) progresses to NASH through various insults causing hepatic injury, ballooning, cell death, inflammation, and consequently progresses to fibrosis (11). Several factors like lipotoxicity, immune response, and gut microbial dysbiosis drive the inflammation in NASH progression (12). Recently, extracellular vesicles involved in cell-cell communications have gained prime attention in the pathogenesis of the disease which is also a promising candidate for biomarkers and therapy in the scenario of limited diagnostic and treatment options in NAFLD (13, 14). Exosomes and microvesicles, the two main populations of extracellular vesicles differing in their size and biogenesis contribute to the pro-inflammatory responses that are critical for the progression of NASH (14). Furthermore, there is growing evidence that extracellular vesicles are also the major contributors in initiating inflammatory responses in liver diseases such as viral hepatitis, alcoholic fatty liver disease, and liver cancer (14–16). This review encapsulates the mechanistic role of inflammatory drivers in NAFLD progression, especially focusing on extracellular vesicles in this cellular crosstalk.

## Lipotoxicity

The intricacy of NAFLD pathobiology is mainly because of multiple parallel hits. Although the root cause for the disease onset is imbalanced fatty acid metabolism, peripheral lipolysis, and elevated *de novo* lipogenesis (DNL), the accumulation of specific toxic lipid intermediates are the main culprits in driving disease progression (17, 18). In addition to non-esterified fatty acids, diacylglycerols, lysophosphatidylcholine, ceramides, and free cholesterol falls under the category of lipotoxic species, which are known to be the critical mediators of inflammation in NAFLD (17, 19). Of note, studies by Yamaguchi et al., have provided experimental evidence that the “quality” of hepatic lipid accumulation plays a key role as opposed to “quantity” of lipids with the risk factor in disease progression and also emphasizes the protective role of triglyceride formation against toxic lipids (20). Along similar lines, while the saturated and trans fatty acids (SFA and TFA) are known to be pro-inflammatory, *in vitro* and *in vivo* models have demonstrated that monounsaturated fatty acids (MUFA) and polyunsaturated fatty acids (PUFA) are cytoprotective aiding in the resolution of inflammation (21–23).

Lipotoxicity leading to mitochondrial and endoplasmic reticulum dysfunction, hepatocellular injury is coupled with activation of apoptotic pathways and inflammasome triggering chronic inflammatory cascades in NASH progression (24–27). Hepatic cell injury is mainly caused by the activation of toll-like receptor 4 (TLR-4), which stimulates TNF-α production *via* the c-Jun N-terminal kinase (JNK) and nuclear factor kappa B (NF-κB) signaling pathways (28). Lipotoxicity causes cell death and acts as a driver of inflammation by activating tumor necrosis factor (TNF)-related apoptosis-inducing ligand receptor 2 (TRAIL-2) and Fas signaling (29). Furthermore, studies by Puri et al., have demonstrated endoplasmic reticulum (ER) as a major contributor in the pathogenesis of NAFLD (24) along with all other studies that underscored the role of oxidative stress (30). Lipotoxic species in extra-hepatic compartments also have predominant pathogenic effects orchestrating metabolic syndrome, gut dysbiosis with NASH progression (31). Recent studies have highlighted the involvement of non-coding RNAs as a mediator of lipotoxic responses especially in TLRs activated by toxic lipids such as palmitate that modulate cellular functions promoting inflammation (32, 33). The extracellular vesicles that are released by the lipotoxic hepatocytes and its role in amplifying inflammation are reviewed in the corresponding section below.

## Cytokines and Chemokines

Relatively innocuous and reversible NAFL that progresses to NASH with the development of hepatic chronic inflammation is characterized by activation of the immune system and several inflammatory cells (34). The activation of immune cells such as macrophages (Kupffer cells), neutrophils, dendritic cells, T-helper cells, B cells, or cytotoxic T cells produces cytokines or chemokines that instigate and drive inflammatory infiltrates in NASH (35). Customary cytokines associated with NAFLD are tumor necrosis factor α (TNF α), transforming growth factor β1 (TGF-β1), various interleukins (IL-6, IL-8, IL-10), and adipocytokines (36). TNF α, released by most immune cells including macrophages, is a first described and one of the major cytokines implicated in NASH evolution (37). Interleukin family cytokines such as IL-6, IL-1β, and IL-18 regulate steatohepatitis progression through hepatic apoptosis, insulin resistance, and induce inflammation *via* activating NF-κB pathways (38–40). Studies by Den Boer et al., and Cintra et al., have demonstrated that deficiency of IL-10, an anti-inflammatory cytokine has a significant role in steatosis and also stimulates the release of proinflammatory cytokines such as TNFα, IL-6, and IL-1β (41, 42). Similarly, adiponectin released from white adipose tissue attenuated inflammation and fibrosis *via* IL-10 by blocking NF-κB inflammatory cascade (43). Besides, chemokines such as monocyte chemoattractant protein 1 (MCP-1)/CCL2, RANTES/CCL5, and other CXC chemokines have gained special importance in the progression of NAFL to NASH by regulating insulin resistance and steatosis *via* the inflammatory crosstalk between liver and adipose tissue (44). Thus, the inflammatory mediators such as cytokines and chemokines (and its receptors) are considered as attractive therapeutic targets in the treatment of NAFLD (45).

## Gut Microbiota

The gut-liver organ crosstalk has been proposed to play a key role in NAFLD pathobiology (23). The gut microbiota exerting beneficial effects over host homeostasis turns out to cause liver damage when there is a perturbation of intestinal microbiota termed as dysbiosis (46). It is reported that various environmental factors such as drugs, diet, host immune system, and the state of intestinal mucosa cause dysbiosis resulting in the formation of leaky gut (47). The increased gut permeability, excess production of endotoxins and ethanol, altered bile acid composition results in the translocation of gut-derived inflammatory products into the liver *via* portal vein (48). Thus intestinal dysbiosis causes systemic inflammation in NASH (49).

Studies in germ-free animal models have revealed the significance of the intestinal microbiome and its role in NAFLD development (50, 51). In addition, studies have shown the host-specific effects of microbial metabolome due to varying individuals’ microbiota, diet, lifestyle, and perinatal milieu (52). Lipopolysaccharides (LPS) activate TLR4 and TLR9 signaling on kupffer cells and induce the production of proinflammatory cytokines and chemokines (53). Furthermore, the inflammasomes (NRLP3 and NRLP6) that recognize damage-associated molecular patterns (DAMPs) derived from toxic lipid species and pathogen-associated molecular proteins (PAMPs) derived from gut microbial products activate the production of IL-18 and IL-1β and promotes liver inflammation (11, 54). Henceforth, it is evident that gut microbiota acts as a driver of inflammation in NAFLD (47, 52).

## Extracellular Vesicles: Inflammatory Crosstalk in NAFLD

Extracellular vesicles (EVs) are non-nucleated, lipid-bound particles secreted by the cells into the extracellular space (55). The study and significance of extracellular vesicles became evident after the work of Harding et al., and Pan and Johnstone, the two independent groups in 1983 (56, 57). Over the last decade, the field of extracellular vesicles has gained prime importance with the discovery of its role as novel mediators in cell signaling. However, the increased scientific publications and dilemma created with the misnomer of extracellular vesicles led to the formation of “The International Society for Extracellular Vesicles” (ISEV). According to the Minimal Information for Studies of Extracellular Vesicles (MISEV) guidelines of 2014 proposed by this society, extracellular vesicles has two subtypes, such as exosomes (30–150 nm in diameter) that are endosome derived and microvesicles (50–1000 nm) or ectosomes, that are derived from the plasma membrane (58). While there exist substantial overlap/no specific protein markers and lack of standardized isolation and analysis methods of extracellular vesicles, the other subtypes can be referred to a) physical characteristics or density of EVs (small EVs, medium/large EVs with specified range); b) biochemical composition (CD81 positive-, CD26 positive-, CD63 positive- EVs); or c) based on the cell of origin (podocyte EVs, Oncosomes, apoptotic bodies) (59, 60).

It is interesting to note that EVs are highly heterogeneous concerning their composition, location, and function as indicated by their parental cells (61). While studies have shown that endosomal sorting complex required for transport (ESCRT)—dependent or—independent machinery is involved in the selection, formation, and grouping of cargos in exosomes (62, 63), the route of microvesicles formation requires cytoskeleton components, molecular motors, and fusion machinery (64). EVs implicated in cell-cell communication are selectively packaged and are enriched with different types of proteins (heat shock proteins—Hsp70, Hsp90; tetraspanins—CD81, CD63, CD9; cytoskeletal proteins—tubulin and actin and other proteins, such as Tsg101, GTPases, Annexins, and cytosolic proteins), lipids (cholesterol, ceramides, sphingomyelin, phosphatidylcholine, and phosphatidylserine), RNAs (messenger RNA, micro RNA, ribosomal RNA, non-coding RNA), and DNA (mitochondrial DNA) as their cargo and can transfer them to other cell types (59, 64). The cargo transfer regulates various cellular activities ranging from gene expression to metabolism (59). Of note, the number of EVs produced and the cargos loaded into these extracellular vesicles depends on the state (physiological or pathological) and microenvironment of the donor cells (65). In this mini-review, we will use the generic term extracellular vesicles (EVs) and decode its role as drivers of inflammation in NAFLD.

**Figure 1:**
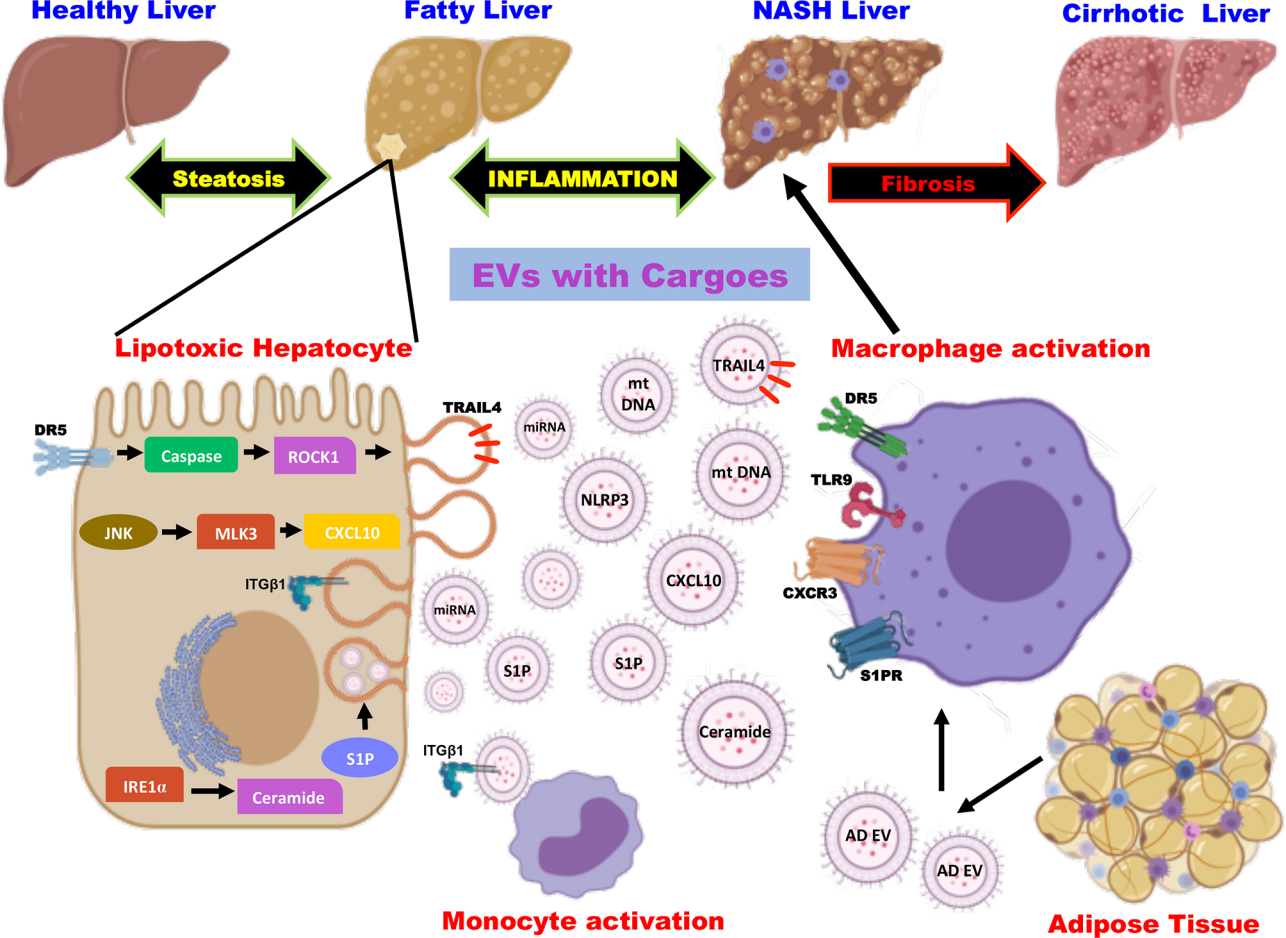
Role of extracellular vesicles as drivers of inflammation in NAFLD. EVs are released from hepatocytes and adipose tissue upon lipotoxic insult and carry cargoes including proteins, lipids, miRNA, and mt DNA. EV cargoes act on the target cells and evoke an inflammatory response by the activation of monocytes and macrophages *via* distinct signaling pathways. EV, extracellular vesicles; miRNA, micro ribonucleic acid; mt DNA, mitochondrial deoxyribonucleic acid; DR5, death receptor 5; ROCK1, rho-associated containing protein kinase 1; JNK, c-Jun N-terminal kinase; MLK3, mixed lineage kinase 3; CXCL10, C-X-C motif ligand 10; IRE1α, inositol-requiring protein-1α; S1P, sphingosine-1-phosphate; TRAIL4, tumor necrosis factor-related apoptosis-inducing ligand 4; NLRP3, nucleotide-binding oligomerization domain-like receptor protein 3; integrin β1 (ITGβ1); AD EV, adipocyte-derived extracellular vesicles; TLR9, toll-like receptor 9; CXCR3, C-X-C motif receptor 3.

The multicellular organ liver with different cell types creates a need for effective intercellular communication to maintain homeostasis. Thus, the cell types of the liver- hepatocytes, kupffer cells, hepatic stellate cells, and natural killer (NK) cells secrete as well as receive the cargos from a plethora of extracellular vesicles (66). The EVs released in basal conditions to meet the normal cell-cell communication seem to increase during various liver diseases including fatty liver diseases (alcoholic fatty liver disease and non-alcoholic fatty liver disease), drug-induced liver injury, viral hepatitis, and liver cancer (16). Studies in high-fat-induced mouse and rat models have shown a significant increase in plasma EV levels, which increase with the NAFLD spectrum (67). It is speculated that hepatocellular stress or injury caused due to lipotoxic lipid species play important role in the elevated release of circulating EVs in a time- and concentration-dependent manner (68). Thus, the released EVs account for processes in NAFLD pathogenesis by promoting inflammation, immune modulation, fibrosis, and angiogenesis (15, 69). Besides hepatocytes, nonparenchymal cell-derived and extra-hepatic origin EVs are also involved in accelerating the disease progression as there exist inter-cellular and inter-organ crosstalk in NAFLD (**Figure 1**). Heterogeneous cargoes with antigen specificity have been identified in these EVs whose actions are dynamic and correlates with histopathological changes in disease (70, 71). In this mini-review, we will discuss mainly the role of EVs and the molecular mechanisms involved in amplifying inflammation by EVs in NAFLD.

Hepatocytes are the major cell types of the liver comprising of 80% of liver volume and hence the hepatocyte-derived EVs play a critical role in the pathogenesis of NAFLD (15, 72). Studies on NASH in human subjects and mouse models have demonstrated that hepatocytes are the major contributors to the increased levels of circulating EVs (69). The mechanistic studies have shown that EVs released from the hepatocytes carrying different types of cargoes act in the local microenvironment *via* distinct cell signaling pathways (**Figure 1**). Kakazu et al., showed that lipotoxic insult to hepatocytes upon treatment with palmitate induces ER stress and activates inositol-requiring protein-1α (IRE1α), protein kinase R-like ER kinase (PERK), and activating transcription factor 6 α (ATF6α) leading to the synthesis of ceramides. These EVs carrying ceramides and its metabolite sphingosine-1-phosphate (S1P) activates macrophages by binding to its receptor (73). Along similar lines, lipotoxicity caused by lysophosphatidylcholine (LPC), a metabolite of palmitate induces the production of EVs loaded with tumor necrosis factor-related apoptosis-inducing ligand (TRAIL) in a death receptor 5 (DR5), caspase and rho-associated containing protein kinase 1 (ROCK1)- dependent manner (74, 75). The TRAIL embedded in the EVs further activates DR5 on the macrophages leading to the production of proinflammatory cytokines such as IL-6 and IL-1β (76). Likewise, inhibition of TRAIL signaling (inhibition of caspase or ROCK1) has been confirmed to be an interesting therapeutic option by improving inflammation, cell injury, and fibrosis (77). Furthermore, Ibrahim et al., have shown that LPC also induces the production of EVs containing C-X-C motif ligand 10 (CXCL10) that activates macrophage chemotaxis in a c-Jun N-terminal kinase (JNK) and mixed lineage kinase 3 (MLK3)-dependent mechanism by both *in vitro* and *in vivo* experiments using MLK3 knock out mouse primary hepatocytes and MLK3 knock out mice, respectively (78).

Studies have also shown that the EVs containing bioactive cargoes like mitochondrial DNA in mice and humans that are implicated in macrophage activation *via* toll-like receptor 9 (TLR-9) serves as an inflammatory signal (79). Cannito et al., have demonstrated that lipotoxic hepatocytes activate the multiprotein platform complex nucleotide-binding oligomerization domain-like receptor protein 3 (NLRP3) inflammasome leading to the production of proinflammatory cytokines (80). On the other hand, cell adhesion molecule termed integrin β1 (ITGβ1) that is released as cargo from EVs of lipotoxic hepatocytes are known to mediate monocyte adhesion to liver sinusoidal endothelial cells and promote inflammation in a mouse model of NASH (81). Recent studies by Jiang et al., described that EVs released by steatotic hepatocytes express high levels of microRNA-1 that mediate proinflammatory signals in endothelial cells by NF-κB activation and Kruppel-like factor 4 (KLF4) downregulation. This seminal work highlights the potential role of hepatocyte-derived EVs in endothelial inflammation leading to atherosclerosis (82). Similarly, the miRNA- let-7e-5p, a component of the EV derived from the hepatocytes is known to enhance lipid accumulation in the adipocytes underscoring the inter organ communication in the pathogenesis of NAFLD (83). It is also noteworthy to mention that EVs containing microRNA 192-5p derived from lipotoxic hepatocytes activate macrophages and increase IL-6 and TNF-α expression and promote inflammation in NAFLD (84).

In addition to promoting inflammation, the EVs released from lipotoxic hepatocytes are also enriched with vanin-1 (VNN), which showed a potent role in angiogenic and fibrogenic signaling pathways in endothelial cells and hepatic stellate cells (HSCs), respectively (85). Various micro RNAs (miR) are found to be an important component of extracellular vesicles leading to inflammation and fibrosis (69). Studies by Lee et al., have demonstrated that upon treatment of lipotoxic palmitate, hepatocytes show elevated levels of EVs (5-fold increase when compared to controls) and microRNAs (miRNA-122 and miRNA-192) that are implicated in driving steatohepatitis to fibrosis by upregulating the expression of genes such as α smooth muscle actin (αSMA), collagen type 1 alpha 1 (col1α1), and transforming growth factor β (TGFβ) in HSC (86). Consistent with this study, elevated levels of miR-122 and miR-192 were observed in mice treated with CDAA (choline-deficient l-amino acid, a diet that causes steatohepatitis) when compared to control diet-fed mice (67) and there also exists a significant positive correlation of these miRNAs with liver enzymes and disease severity in NAFLD patients (87).

As mentioned previously, in NASH, EV-mediated intercellular and interorgan crosstalk represents central events in the progression of the disease. Besides hepatocyte-derived EVs, the EVs derived from other non-parenchymal cells also play a pivotal role in the pathogenesis of NASH (69, 88). While EVs from cholangiocytes are known to induce angiogenic signaling in liver sinusoidal endothelial cells, studies by Wang et al., have reported that EVs derived from the endothelial cells contribute to liver fibrosis *via* sphingosine kinase 1 (SK1) (89, 86). Furthermore, profibrogenic signaling is also activated in HSCs leading to liver fibrosis (90). Consistently, Kornek et al., revealed experimental data for the presence of EVs carrying CD4⁺ and CD8⁺ T cells, NK cells in the plasma of NAFLD patients (91). Adipocyte-derived EVs exert a crucial role in augmenting insulin resistance and triggering hepatic inflammation *via* MCP-1 and IL-6 (92). Notably, Thomou et al., have provided evidence that adipose tissue serves as a major source of EVs carrying micro RNA using mice lacking dicer in adipose tissue (93).

Currently, NAFLD has no efficient technique of diagnosis for staging the disease except for the highly invasive gold standard technique of liver biopsy. Multiple non-invasive techniques have been practiced to assess NAFLD severity such as analyzing serum levels of liver enzymes, ultrasound-based elastography, and magnetic resonance imaging (MRI) (94). However, these techniques lack proper efficacy and precision in early diagnosis, severity, and grading of disease stage (95). The implication of EVs in the pathobiology of different stages in NAFLD spectra has made it a potent biomarker in NAFLD diagnosis (15, 96). Due to their availability in biological fluids such as blood and ease of intercellular communication, EVs are proposed to be an excellent non-invasive liquid biomarker referred to as “liquid biopsy”. There are several EV biomarkers (protein-based, lipid-based, or nucleic acid-based EV biomarkers) that have been documented and it is known that the abundance and molecular composition of these circulating EVs vary and signify different disease conditions which would help in improving the current status of diagnosis of NAFLD (97–99). Importantly, EVs have emerged as a therapeutic tool in treating NAFLD due to their ability in stable delivery of drugs, miRNA, siRNA, or other cargoes and easy uptake by the liver cells paving the path to hope towards EV-based therapy. Additionally, studies have also demonstrated EVs as a potential therapeutic target as inhibition of EV-mediated pathological processes can ameliorate disease progression in NAFLD (78, 89).

## Conclusions

NAFLD is a multifactorial complex diseased condition caused by multiple parallel hits and covers a wide spectrum of liver damage. Accumulation of toxic lipid species linked with inter-organ crosstalk of adipose tissue-liver axis and gut-liver axis contributes to the activation of the innate immune system and inflammatory signals through several cytokines and chemokines. Developed over the last decades is the idea of crosstalk in the pathogenesis of NASH *via* circulating extracellular vesicles. Many studies conducted over this period have revealed the intricate role of EVs in driving several features of NASH such as inflammation, innate immune response, profibrogenic activation most of which are rooted in lipotoxicity. Thus, EVs are important molecular entities involved as mediators in cell-cell and organ-organ communication contributing to liver physiology and pathophysiology.

Exploiting the attractive and easy circulating nature of EVs, several of them have been characterized as a potential biomarker candidate. Given the significance, upon further critical evaluation of these EVs, it is likely to emerge as a promising biomarker for easy diagnosis, assessing the severity and stages of the disease. However, there still remains an uphill battle in terms of characterizing and investigating the EVs due to their heterogeneity and differential expression in various diseases. Similarly, the feasibility of blockade of EV secretion as a therapeutic strategy is far beyond implication, as it demands a further understanding of the role of EVs in normal physiology. Active research and collaboration between scientists and physicians would pay a way for the successful transition of current EV knowledge from bench to bedside. In conclusion, the comprehensive investigation leading to a better understanding of the regulation and mechanisms of EV biogenesis and secretion and functional analysis of its associated cargoes would aid in filling the much-needed lacuna in the diagnosis and therapy of NAFLD.

## Author Contributions

AS and DK devised and wrote the manuscript. DiS and DK made the figures. PS and DeS provided input and edited the manuscript. All authors contributed to the article and approved the submitted version.

## Funding

This study was supported by Ramalingaswami Re-entry Fellowship to DPK from the Department of Biotechnology (DBT), Government of India.

## Conflict of Interest

The authors declare that the research was conducted in the absence of any commercial or financial relationships that could be construed as a potential conflict of interest.
